# Mitochondrial and microbial diversity of the invasive mosquito vector species *Culex tritaeniorhynchus* across its extensive inter-continental geographic range

**DOI:** 10.12688/wellcomeopenres.20761.1

**Published:** 2024-01-12

**Authors:** Claire L. Jeffries, Luciano M Tantely, Perparim Kadriaj, Marcus S C Blagrove, Ioanna Lytra, James Orsborne, Hasan Mohammad Al-Amin, Abdul Rahim Mohammed, Mohammad Shafiul Alam, Romain Girod, Yaw A Afrane, Silvia Bino, Vincent Robert, Sebastien Boyer, Matthew Baylis, Enkelejda Velo, Grant L Hughes, Thomas Walker

**Affiliations:** 1 Department of Disease Control, Faculty of Infectious and Tropical Diseases, London School of Hygiene and Tropical Medicine, London, WC1E 7HT, UK; 2 Unite d'entomologie medicale, Institute Pasteur de Madagascar, Antanarivo, Madagascar; 3 Vector Control Unit, Control of Infectious Diseases Department, Institute of Public Health, Tirana, Albania; 4 Department of Evolution, Ecology and Behaviour, Institute of Infection, Veterinary and Ecological Sciences, University of Liverpool, Liverpool, England, UK; 5 Health Protection Research Unit on Emerging and Zoonotic Infections, University of Liverpool, Liverpool, England, UK; 6 Department of Entomology and Agricultural Zoology, Benaki Phytopathological Institute, Athens, Greece; 7 Infectious Diseases Division, International Centre for Diarrhoeal Disease Research, Dhaka, Bangladesh; 8 Berghofer Medical Research Institute, Queensland Institute of Medical Research, Brisbane, Australia; 9 Department of Medical Microbiology, University of Ghana Medical School, University of Ghana, Accra, Greater Accra Region, Ghana; 10 MIVEGEC, CNRS, Institute of Research for Development (IRD), University of Montpellier, Montpellier, France; 11 Medical and Veterinary Entomology Unit, Institut Pasteur du Cambodge, Phnom Penh, Cambodia; 12 Department of Livestock and One Health, Institute of Infection, Veterinary and Ecological Sciences, University of Liverpool, Liverpool, England, UK; 13 Departments of Vector Biology and Tropical Disease Biology, Centre for Neglected Tropical Disease, University of Liverpool, Liverpool, England, UK; 14 School of Life Sciences, University of Warwick, Coventry, England, UK

**Keywords:** mosquitoes, Wolbachia, arboviruses, microbiome

## Abstract

**Background:**

*Culex (Cx.) tritaeniorhynchus* is an invasive mosquito species with an extensive and expanding inter-continental distribution, currently reported across Asia, Africa, the Middle East, Europe and now Australia. It is an important vector of medical and veterinary pathogens which cause significant morbidity and mortality in human and animal populations. Across regions endemic for Japanese encephalitis virus (JEV), *Cx. tritaeniorhynchus* is considered the major vector and has also been shown to contribute to the transmission of several other zoonotic arboviruses including Rift Valley fever virus (RVFV) and West Nile virus (WNV).

**Methods:**

In this study, we used laboratory vector competence experiments to determine if *Cx. tritaeniorhynchus* from a Southern European population were competent JEV vectors. We also obtained samples from multiple geographically dispersed *Cx. tritaeniorhynchus* populations from countries within Europe, Africa, Eurasia and Asia to perform phylogenetic analysis to measure the level of mitochondrial divergence using the *cytochrome oxidase subunit 1* ( *CO1*) gene. We also undertook bacterial *16S rRNA* gene amplicon sequencing to determine microbial diversity and used multi-locus sequence typing (MLST) to determine any evidence for the presence of strains of the naturally occurring endosymbiotic bacterium *Wolbachia*.

**Results:**

*Cx. tritaeniorhynchus* from a Greek population were shown be be competent vectors of JEV with high levels of virus present in saliva. We found a signficant level of mitochondrial genetic diversity using the mosquito *CO1* gene between geographically dispersed populations. Furthermore, we report diverse microbiomes identified by *16S rRNA* gene amplicon sequencing within and between geographical populations. Evidence for the detection of the endosymbiotic bacteria *Wolbachia* was confirmed using *Wolbachia*-specific PCR and MLST.

**Conclusions:**

This study enhances our understanding of the diversity of *Cx. tritaeniorhynchus* and the associated microbiome across its inter-continental range and highlights the need for greater surveillance of this invasive vector species in Europe.

## Plain Language Summary

The mosquito species *Culex (Cx.) tritaeniorhynchus* is expanding its range and is now present in over 50 countries across Asia, Africa, the Middle East, Europe and now Australasia. It can transmit human and animal pathogens, resulting in significant morbidity and mortality. This species transmits Japanese encephalitis virus in endemic areas of Asia, and it has also been shown to contribute to the transmission of several other viruses that can infect humans, including Rift Valley fever virus and West Nile virus. In this study, we firstly undertook some lab experiments to show that *Cx. tritaeniorhynchus* from a Southern European population are competent vectors of Japanese encephalitis virus. We also obtained field mosquitoes from countries within Europe, Africa, Eurasia and Asia and used phylogenetic analysis to demonstrate a high level of mitochondrial divergence within and between populations. In addition, we analysed the bacteria present within mosquitoes and found a high level of microbial diversity. Finally, we present evidence for the presence of *Wolbachia* endosymbiotic bacteria in some populations of this mosquito species. This study highlights the need for greater surveillance of this invasive vector species – particularly in Europe.

## Introduction

The invasive mosquito vector species *Culex (Cx.) tritaeniorhynchus* has a wide and expansive distribution which includes populations in over 50 countries. Ranging across Asia, the Middle East and Africa ( Jeffries & Walker, 2015), in recent decades it has been reported in Europe ( Samanidou & Harbach, 2003), Eurasia ( Gugushvili, 2002), Cape Verde off western Africa ( Alves *et al*., 2014) and was recently recorded for the first time in Australia ( Lessard *et al*., 2021). *Cx. tritaeniorhynchus* is the major vector of Japanese encephalitis virus (JEV) in Asia and the Pacific ( Jeffries & Walker, 2015) and resulting JEV transmission leads to an estimated 50,000 – 175,000 cases of human disease annually ( Campbell *et al*., 2011; Erlanger *et al*., 2009). Estimates suggest JE clinical disease presentations account for only 1% of total viral infections, indicating overall occurrence of human JEV infections could be in the region of 5 – 17.5 million each year, with almost four billion people living in 24 endemic countries at risk ( Jeffries & Walker, 2015; Weaver & Reisen, 2010). There have also been recent cases of JEV detection in mosquitoes and birds in Italy ( Platonov *et al*., 2012; Ravanini *et al*., 2012) and an autochthonous human case in Angola, Africa ( Simon-Loriere *et al*., 2017), in addition to the recent JEV outbreak in Australia, highlighting the possibilities of future viral spread and establishment in novel regions and naïve populations ( Gao *et al*., 2019; Lord, 2021; Weaver & Reisen, 2010). *Cx. tritaeniorhynchus* is also capable of transmitting several other significant zoonotic arboviruses including RVFV ( Jupp *et al*., 2002) and WNV ( Akhter *et al*., 1982; Fontenille, 1989; Reisen *et al*., 1982; Turell *et al*., 2006) with viral geographic distributions extensively overlapping with the current range of *Cx. tritaeniorhynchus*.

The widespread presence of this invasive species warrants further investigation to determine if outbreaks could occur beyond the current arboviral transmission zones. *Cx. tritaeniorhynchus* is well established in Southern European countries such as Greece and Albania but the capacity to transmit JEV has not been assessed to date. Although laboratory vector competence studies can measure the capability and efficiency of a mosquito vector population to transmit arboviruses under experimental conditions, the vectorial capacity in wild populations is influenced by multiple factors, including genetic diversity and the composition of the microbiota ( Bolling *et al*., 2015; Cansado-Utrilla *et al*., 2021; Franz *et al*., 2015; Jupatanakul *et al*., 2014; Kilpatrick *et al*., 2010; Takken & Verhulst, 2013). Although several studies have investigated genetic diversity and vector competency in *Cx. tritaeniorhynchus*, they were mostly confined to populations within the same region or country - largely within the Asian continent ( Ashfaq *et al*., 2014; Hayes *et al*., 1984; Luo *et al*., 2016; Philip Samuel *et al*., 2010; Sakai & Baker, 1972; Takahashi, 1980; Turell *et al*., 2006; Xie *et al*., 2021).

Mosquito vectors have associations with a wide diversity of microbes ( Jupatanakul *et al*., 2014), including bacteria such as *Wolbachia, Asaia*, *Serratia* and *Pseudomonas,* which can affect vectorial capacity ( Bolling *et al*., 2015; Minard *et al*., 2013). Some studies on the native microbiome or associated bacteria have been carried out in *Cx. tritaeniorhynchus* ( Guo *et al*., 2016; Kittayapong *et al*., 2000; Tsai *et al*., 2004), but cross population microbiome composition studies comparing within and between geographically dispered populations has not been extenstive studied. In this study, we undertook laboratory vector competence experiments using an early generation colony of *Cx. tritaeniorhynchus* originating from Greece to demonstrate that European populations are competent vectors of JEV. We also obtained geographically dispersed *Cx. tritaeniorhynchus* from multiple populations (spanning four continents) to examine the mitochondrial and microbial diversity of this invasive and medically important vector species. Our results show evidence for mitochondrial divergence, a high level of microbial diversity and the presence of the endosymbiotic bacterium *Wolbachia* at only low prevalence and in only some geographic populations.

## Methods

### European population colonisation and JEV vector competence experiments

Field-collected *Cx. tritaeniorhynchus* larvae (~500) from Messolonghi, Greece were transported to the London School of Hygiene and Tropical Medicine for initiation of a colony. A range of techniques were employed to optimise insectary conditions for all mosquito life stages including large rearing cages (110 × 55 × 55 cm), light cycles with dusk/dawn simulation and swarm markers. JEV vector competence was assessed on the fourth generation at the Liverpool School of Tropical Medicine. Blood meals (heparinised human blood, NHS transfusion service, Speke) containing JEV (strain CNS138-11), to a final concentration of 6 log 10 plaque-forming units/mL, were provided for three hours, using a Hemotek membrane feeding system and an odourised feeding membrane, to 5–7-day-old adult females from which sugar sources had been withheld for 24 hours. Blood-fed females were incubated at 27°C, 70% humidity, for 14 days prior to collection of saliva using a forced salivation technique ( Moreira *et al*., 2009). The head/thorax and abdomen were separated for surviving females after the 14-day incubation and the dissected body parts were stored for RNA preservation. RNA was extracted using Qiagen RNeasy kits from all saliva and body-part samples and tested by JEV-specific quantitative RT-PCR analysis ( Yang *et al*., 2004) to determine infection rates. qPCR reactions were prepared using 5 μL of FastStart SYBR Green Master mix (Roche Diagnostics) with a final concentration of 1μM of each primer, 1 μL of PCR grade water and 2 μL template DNA, to a final reaction volume of 10 μL. Prepared reactions were run on a Roche LightCycler 96 System and amplification was followed by a dissociation curve (95°C for 10 s, 65°C for 60 s and 97°C for 1 s) to ensure the correct target sequence in addition to the inclusion of positive controls (RNA extracted from JEV strain CNS138-11) and no template controls (NTCs).

### Mosquito field collections

**Figure 1.**
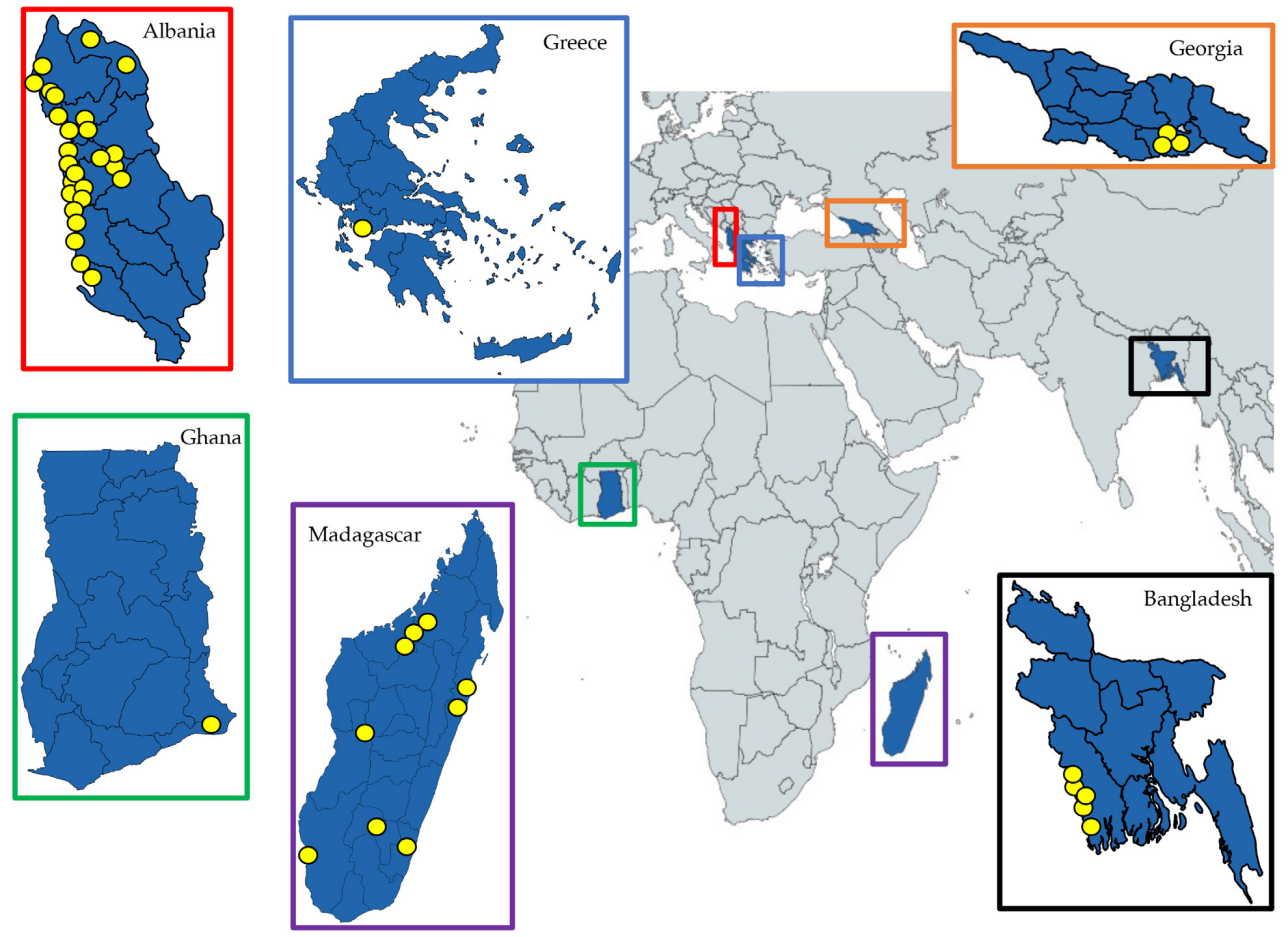
Locations of collection sites for *Cx. tritaeniorhynchus* samples analysed in this study. Yellow dots represent estimated collection locations within the six countries in our study (GPS co-ordinates, collection methods and number of specimens are shown in Table 1). Maps were produced using Mapchart licensed under a Creative Commons Attribution-ShareAlike 4.0 International License.

*Culex tritaeniorhynchus* specimens were obtained from field-collections in Albania, Greece, Georgia, Ghana, Madagascar and Bangladesh ( Figure 1). The locations, year, GPS co-ordinates and number of specimens obtained are shown in Table 1. Specimens were collected in Albania during country-wide entomological surveys in 2015–2016, in addition to focused field-work collections in 2017, including in the rural village of Sop in the Fier district, south-western Albania. A variety of adult trapping techniques and larval dipping were used. Specimens were preserved in Invitrogen RNAlater at -20°C followed by -70°C long-term storage. Field-collected fourth instar larvae were obtained from three sites in south-eastern Georgia in September 2015; using larval dipping in semi-permanent water bodies with vegetation, then stored in 70% ethanol. In Ghana, specimens were collected as adults from Dogo village in the Greater Accra region in June 2017 ( Orsborne *et al*., 2019). Adult specimens were preserved in Invitrogen RNAlater with cold storage. Collections in Madagascar were carried out in 2015–2016, from locations spanning various bioclimatic ecotype zones ( Jeffries *et al*., 2018b), and specimens were preserved in RNAlater with cold storage to prevent RNA degradation. Adult collections in Bangladesh were carried out in Sept–Nov 2013 from five sites within two districts in the Rajshahi Division in western Bangladesh. Within the district of Rajshahi, mosquitoes were collected from the upazilas (sub-districts) of Paba, Puthia and Bagmara, and within the Naogaon district, specimens were obtained from the upazilas of Manda and Mohadevpur. Samples were stored dry with silica gel desiccant to prevent microbial growth.

**Table 1.** *Cx. tritaeniorhynchus* collection locations and samples collected. Latitude and longitude coordinates are in decimal degrees. NBFF = non-blood-fed females, BFF = blood-fed females, *denotes from field-collected larvae.

Country	Year, Location (latitude, longitude)	*Cx. tritaeniorhynchus* samples					
		Larvae	Adults *	NBFF	BFF	Males	Total
Albania	2015, Fier (40.724,19.556)	-	-	263	6	-	269
	2016, Fier (40.724,19.556)	-	30	546	28	1	605
	2017, Fier (40.724,19.556)	613	24	675	288	28	1628
	2015, Lezhe (41.784,19.644)	-	16	2	4	-	22
	2016, Lezhe (41.784,19.529)	-	-	4	-	-	4
	2016, Lushnje (40.997,19.529)	-	-	1	-	-	1
	2015, Sarande (39.876,20.005)	-	-	2	1	1	4
	2016, Sarande (39.876,20.005)	-	-	1	-	-	1
	2016, Shkoder (42.068,19.513)	-	-	2	-	-	2
	2015, Vlore (40.467,19.490)	-	-	-	-	3	3
	2016, Vlore (40.467,19.490)	-	-	16	-	-	16
Greece	2014, Messolonghi (38.339,21.252)	500	60	-	-	-	560
Georgia	2015, Tsereteli (41.423, 44.824)	2	-	-	-	-	2
	2015, Gordabani (41.464, 45.099)	5	-	-	-	-	5
	2015, Mzianeti (41.474, 45.165)	3	-	-	-	-	3
Bangladesh	2013, Paba (24.378, 88.533)	-	-	10	1	-	11
	2013, Puthia (24.405, 88.888)	-	-	1	2	-	3
	2013, Bagmara (24.602, 88.900)	-	-	7	7	-	14
	2013, Mohadevpur (24.939, 88.718)	-	-	-	8	-	8
	2013, Manda (24.801, 88.749)	-	-	2	5	-	7
Madagascar	2015, Brickaville (-18.824, 49.077)	-	-	10	-	-	10
	2015, Farafangana (-22.821, 47.819)	-	-	9	1	-	10
	2015, Ihosy (-22.412, 46.129)	-	-	10	-	-	10
	2015, Maevatanana (-17.027, 46.767)	-	-	9	1	-	10
	2015, Mampikony (-16.100, 47.632)	-	-	1	29	-	30
	2015, Miandrivazo (-19.533, 45.449)	-	-	9	1	-	10
	2015, Toamasina (-18.148, 49.404)	-	-	10	-	-	10
	2015, Tulear (-23.387, 43.717)	-	-	-	32	11	43
	2016, Tsaramandroso (-16.367, 46.993)	-	-	14	23	6	43
Ghana	2017, Dogo (5.874, 0.560)	-	-	7	-	-	7

### Morphological identification

Adult specimens were morphologically identified using keys appropriate for the geographic region ( Günay *et al*., 2017; Lytra & Emmanouel, 2014b; Reuben *et al*., 1994; Robert *et al*., 2019; Samanidou & Harbach, 2003; Tantely *et al*., 2016), observing distinctive morphological features, including the presence of a clear white band on the proboscis, entirely dark wings and ringed tarsi ( Samanidou & Harbach, 2003). Adult female mosquito physiological status was recorded and if wild-caught blood-fed, then the stage of digestion and time since blood-feeding was approximated using the Sella score method ( Detinova, 1962; Martínez-de la Puente *et al*., 2013).

### Nucleic acid extraction and molecular species confirmation

Dependent upon the collection location, techniques and preservation methods, the relevant nucleic acid extraction methods were undertaken. All specimens were homogenised using a Qiagen Tissue Lyser II and Qiagen 3mm stainless steel beads. DNA was extracted from specimens using a Qiagen DNeasy Blood and Tissue kit according to the manufacturer’s instructions. Some samples where subject to RNA extraction alone, or a modified method for simultaneous RNA and DNA co-extraction utilising Trizol reagent extraction protocol, prior to column-based extraction of the relevant phase using Qiagen DNeasy or RNeasy kits. RNA eluates were converted to cDNA using Applied Biosystems High Capacity cDNA Reverse Transcription kits. To confirm morphological species identification, gDNA or cDNA was used in broad-specificity barcoding PCRs, followed by Sanger sequencing and phylogenetic analysis (as detailed for mitochodrial diversity analysis below) to confirm species identification.

### Intra- and inter-population mitochondrial diversity

Mitochodrial diversity was assessed through Sanger sequencing of amplified PCR products from multiple assays targeting the *cytochrome oxidase subunit 1* ( *CO1*) gene ( Bernasconi *et al*., 2000; Folmer *et al*., 1994; Kumar *et al*., 2007; Zittra *et al*., 2016) depending on the geographic region. Sub-sample testing was used to select a primer set ( Kumar *et al*., 2007) producing a ~700 base pair (bp) product for screening all geographical populations. A primer combination to amplify the full length *CO1* gene, binding at the 5’ and 3’ tRNA respectively, produced a ~1150bp sequence ( Bernasconi *et al*., 2000; Shaikevich & Zakharov, 2010; Simon *et al*., 1994).

### Consensus sequence and alignment assembly

**Figure 2.**
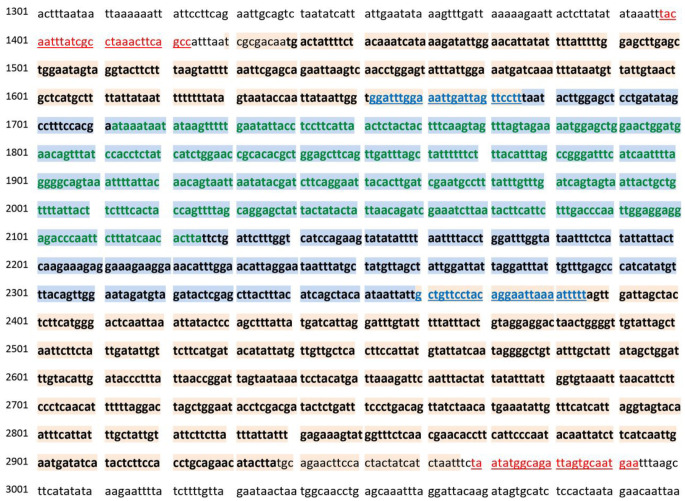
*CO1* primer sets and sequence alignment positions. Excerpt of nucleotide sequence from the complete mitochondrial reference genome of *Cx. tritaeniorhynchus* (NC_028616). Red, underlined text – primer binding sites o background – nucleotides comprising the full *CO1* gene; Blue, underlined – Kumar *et al.* primer binding sites; Blue background – Position of alignment (a) of sequence set binding regions; Text in bold – Location of alignment (b) maximizing the length and with almost full *CO1* gene coverage; Green text – Location of alignment (c) maxi included.

Sequencing analysis was carried out in MEGA11 ( Tamura *et al*., 2021) as previously described ( Walker *et al*., 2021). All mitochondrial *CO1* nucleotide sequences for *Cx. tritaeniorhynchus* available on GenBank (NCBI: txid7178, 992 sequences) were downloaded and aligned with the 69 *CO1* sequences generated in this study. Three *CO1* alignments were constructed; (A) all *Cx. tritaeniorhynchus* sequences with coverage of the fragment generated by the Kumar *et al.* primer set ( Kumar *et al*., 2007) (253 sequences, 686 positions) including concomitant species obtained during field-collections, (B) all *Cx. tritaeniorhynchus CO1* gene full length sequences, maximising the length (20 sequences, 1538 positions), and (C) all *Cx. tritaeniorhynchus CO1* sequences currently available with sufficient fragment overlap, to balance the length of alignment but maximise number of reference sequences included (1007 sequences, 414 positions). The positions of the alignments and primer binding regions according to the *Cx. tritaeniorhynchus* complete mitochondrial genome reference sequence NC_028616 is shown in Figure 2.

### Phylogenetic tree construction and analysis

Each alignment was examined using the “Find-Best-Fit Maximum Likelihood substitution model” for phylogenetic analysis and tree construction as previously described ( Jeffries *et al*., 2021). Models used were the General Time Reversible model ( Nei & Kumar, 2000) (GTR) or the Tamura three-parameter model ( Tamura, 1992) (T92). The tree with the highest log likelihood is shown. The percentage of trees in which the associated taxa clustered together is shown next to the branches. Initial tree(s) for the heuristic search were obtained automatically by applying Neighbor-Joining and BioNJ algorithms to a matrix of pairwise distances estimated using the Maximum Composite Likelihood (MCL) approach, and then selecting the topology with superior log likelihood value. The trees are drawn to scale, with branch lengths measured in the number of substitutions per site. Codon positions included were 1st+2nd+3rd+Noncoding. All positions containing gaps and missing data were eliminated. The phylogeny test was by Bootstrap method with 1000 replications. Evolutionary analyses were conducted in MEGA11 ( Tamura *et al*., 2021).

### Mitochondrial diversity and haplotype analyses

Mitochondrial diversity of populations was further assessed through mitochondrial diversity metrics, analysis of haplotypes, generation of haplotype networks, and pairwise comparison of genetic differentiation, including both study-generated and available reference *CO1* sequences. *Cx. tritaeniorhynchus CO1* alignments were analysed using DnaSP V6.12.03 ( Rozas *et al*., 2017) to assess sequence polymorphisms and determine nucleotide and haplotype diversity. Haplotype networks were constructed within PopART ( Leigh & Bryant, 2015) using the TCS inference method ( Clement *et al*., 2000). Intra- and inter-group variation was assessed at the individual, country and regional population levels using Arlequin V3.5.2.2 ( Excoffier & Lischer, 2010), with analysis of molecular variance (AMOVA) ( Excoffier *et al*., 1992) and visualisation of outputs in R V3.5.0 ( R Core Team, 2018).

### Bacterial *16S rRNA* gene amplicon sequencing

Microbiomes of selected specimens were analysed using barcoded high-throughput amplicon sequencing of the bacterial *16S rRNA* gene. To enable analysis of the differences in microbiome between species ( *Cx. tritaeniorhynchus* and concomitant species), physiological status (blood-fed or non-blood-fed) and geographic location (both intra- and inter-country) samples were selected from specific groups for comparison ( Table 2). Mosquito specimens were surface sterilised prior to extraction, and negative controls comprising both DNA extraction and RNA extraction–Reverse Transcription blanks were included alongside the samples. Sequencing of each extract was undertaken using universal *16S rRNA* V3-V4 region primers (FOR: 5’-CCTACGGGNGGCWGCAG-3’, REV: 5’-GGACTACHVGGGTATCTAATCC-3’) ( Klindworth *et al*., 2013) in accordance with standard Illumina *16S rRNA* metagenomic sequencing library protocols with the Nextera XT Index Kit v2 used to barcode samples for multiplexing. Sequencing was performed on an Illumina MiSeq, with the MiSeq v2 (500 cycle) reagent kit, with libraries sequenced as 250bp paired-end reads (PE).

**Table 2.** Sampling groups and associated information for *16S* microbiome analysis. Abbreviations: N = Number of samples in that group, NBFF = Non-blood-fed-female, BFF = Blood-fed-female, NA type = Nucleic Acid type, gDNA = genomic DNA, cDNA = complementary DNA (RNA extracts after reverse-transcription).

Group	Species	Country	Collection Location	N	Status	Body part	NA type
AA	*Cx. tritaeniorhynchus*	Bangladesh	Paba, Rajashahi	10	NBFF	Whole	gDNA
AB	*Cx. tritaeniorhynchus*	Bangladesh	Bagmara, Rajashahi	7	NBFF	Whole	gDNA
B	*Cx. tritaeniorhynchus*	Albania	Fier (Sop)	16	NBFF	Whole	gDNA
C	*Cx. pipiens*	Albania	Fier (Sop)	16	NBFF	Whole	gDNA
D	*Oc. caspius*	Albania	Fier (Sop)	16	NBFF	Whole	gDNA
E	*Cx. tritaeniorhynchus*	Albania	Fier (Sop)	15	NBFF	Abdomen	cDNA
F	*Cx. tritaeniorhynchus*	Albania	Fier (Sop)	12	BFF	Abdomen	cDNA
G	*Cx. tritaeniorhynchus*	Albania	Vlore	10	NBFF	Abdomen	cDNA
H	*Cx. tritaeniorhynchus*	Madagascar	Brickaville	10	NBFF	Whole	cDNA
I	*Cx. tritaeniorhynchus*	Madagascar	Farafangana	9	NBFF	Whole	cDNA
J	*Cx. tritaeniorhynchus*	Madagascar	Ihosy	10	NBFF	Whole	cDNA
K	*Cx. tritaeniorhynchus*	Madagascar	Maevatanana	9	NBFF	Whole	cDNA
L	*Cx. tritaeniorhynchus*	Madagascar	Miandrivazo	9	NBFF	Whole	cDNA
M	*Cx. tritaeniorhynchus*	Madagascar	Toamasina	10	NBFF	Whole	cDNA
N	*Cx. tritaeniorhynchus*	Madagascar	Tsaramandroso	12	NBFF	Abdomen	cDNA
O	*Cx. tritaeniorhynchus*	Madagascar	Tsaramandroso	15	BFF	Abdomen	cDNA
P	*Cx. antennatus*	Madagascar	Tsaramandroso	14	NBFF	Abdomen	cDNA

### Data cleaning, quality control and filtering

Microbiome bioinformatics analyses were carried out on demultiplexed reads using “Quantitative Insights Into Microbial Ecology” (QIIME)2 Core (q2cli) 2020.2 distribution ( Bolyen *et al*., 2019). Demultiplexed reads were divided according to extract type of gDNA ( *16S* of all microbiota present) or cDNA (actively expressed microbial *16S*) (along with their respective blank control samples) and analysed separately in downstream analysis. Reads were imported into QIIME2 and the V3-V4 primer and Nextera adapter sequences were removed using the “q2-cutadapt” plugin ( Martin, 2011). Quality plots were generated and visualised using the “q2-demux summarize” command to assess and select optimal quality filtering parameters including truncation length for any adaptor sequence removal. Quality filtering, denoising and chimera removal was carried out using the “Diversive Amplicon Denoising Algorithm” (DADA) “q2-dada2” plugin ( Callahan *et al*., 2016) (“denoise-paired” command, gDNA: “p-trunc-len-f 231, p-trunc-len-r 229”; cDNA: “p-trunc-len-f 233, p-trunc-len-r 229”) to group Amplicon Sequence Variants (ASVs) within the data. The feature-table artifacts generated were filtered to exclude features present within the blank controls (“q2-feature-table filter-samples”).

### Taxonomic identification of features

Taxonomic assignment of ASVs was carried out using the “q2-feature-classifier” plugin ( Bokulich *et al*., 2018) (“classify-sklearn” command ( Pedregosa *et al*., 2011)) with a pre-trained SILVA classifier (Naive Bayes classifier was pre-trained on the *16S rRNA* SILVA SSU v138 99% reference database ( Quast *et al*., 2013), with the V3-V4 primers). The taxonomy classifier generated was used to remove mitochondrial and chloroplast ASVs from each feature table (“q2-taxa filter-table” plugin) to remove host and background non-relevant features. The samples were then filtered further (“q2-feature-table filter-features”) to remove features with frequencies below 100, and to only include the relevant samples for each comparative analysis. The taxonomic assignments were visualised using “q2-taxa barplot” to show relative taxonomic abundance across all individual samples.

### Alpha and beta diversity analysis

Within the qiime2 phylogeny plugin, the “q2-phylogeny align-to-tree-mafft-fasttree” command was used, incorporating representative sequence artifacts from each of the gDNA and cDNA groups (rep-seqs output from DADA2) to produce rooted phylogenetic trees for diversity analysis. For each comparison set, using the respective filtered feature tables, alpha and beta diversity analysis was conducted through the qiime2 diversity plugin, using “qiime diversity core-metrics-phylogenetic” ( Faith, 1992). The sampling depth was selected from visualising feature tables (“q2-feature-table summarize”) for each comparison, generating alpha rarefaction visualisations (“q2-diversity alpha-rarefaction”) with “-p-max-depth” just over the median frequency per sample from the feature table, and then by balancing the number of features, with the number of samples from each group retained ( Weiss *et al*., 2017). The diversity core metrics results were then generated and visualised using the relevant alpha or beta “group-significance” commands ( Anderson, 2001; Kruskal & Wallis, 1952). Pairwise PERMANOVA tests with 999 permutations were used for comparisons between groups for the variable of interest and a significance level of P value <0.01 was used as the threshold. The metrics consulted for alpha (within-group) diversity were the Shannon diversity Index, Faith’s phylogenetic diversity and the Evenness. For beta (between-group) diversity the metrics consulted were the Bray-Curtis, Unweighted-Unifrac (pairwise) and Weighted-Unifrac.

### Differential abundance testing – ANCOM

To test for the presence of any differentially abundant taxa within each sample comparison group the analysis of composition of microbiomes (ANCOM) method was used within the qiime2 composition plugin ( Mandal *et al*., 2015). The “q2-composition add-pseudocount” command was used, followed by “q2-composition ancom” with the relevant variable selected for each comparison, to investigate if any association may be apparent. Results were visualised in volcano plots, and assessed through the test statistic, W, to determine significance.

### *Wolbachia* PCR detection and multi-locus strain typing

Amplification of *Wolbachia*-specific 16S rRNA gene sequences was undertaken targeting the conserved *Wolbachia* 16S rRNA gene using W-Spec primers (438bp) ( Werren & Windsor, 2000), in addition to a primer set designed for quantitiave PCR (target length: 102bp) ( Gomes *et al*., 2017). *Wolbachia* MLST was undertaken to characterise *Wolbachia* strains using the sequences of five conserved genes ( *gatB*, *coxA*, *hcpA*, *ftsZ* and *fbpA*) as molecular markers to genotype each strain ( Baldo *et al*., 2006). In addition, an alternative primer set targeting a 271bp fragment of the ftsZ gene sequence in *Wolbachia* strains from Supergroups A and B was used on selected samples ( de Oliveira *et al*., 2015). PCR reactions and Sanger sequencing of *Wolbachia* MLST PCR products were carried out as previously described ( Jeffries *et al*., 2018a). Sequencing analysis was carried out in MEGA11 ( Tamura *et al*., 2021), using methodology previously described, with consensus sequences used to perform nucleotide BLAST (NCBI) database queries, and for *Wolbachia* gene searches against the *Wolbachia* MLST database ( http://pubmlst.org/wolbachia). Phylogenetic analysis of MLST gene locus sequences was performed as previously described for the mosquito *CO1* gene.

## Results

### JEV vector competence of a *Cx. tritaeniorhynchus* Greek population

**Figure 3.**
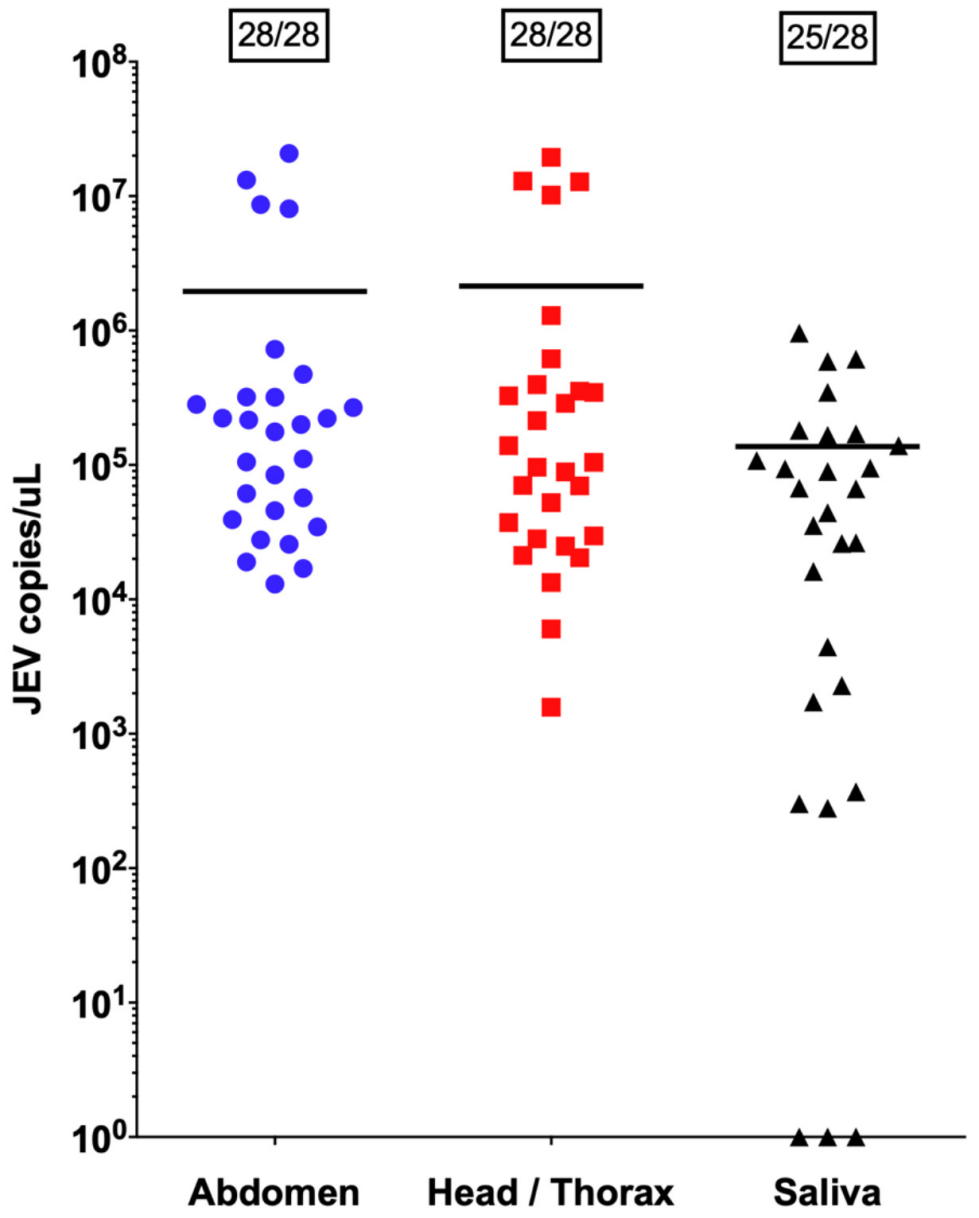
JEV vector competence experiment on European *Cx. tritaeniorhynchus* colonised from Greece. Scatter dot plot of quantitative PCR data. Horizontal bars represent mean JEV copies/µl per group. Boxed numbers show the number of JEV positive samples / total number of samples tested per group.

After incubation for 14 days following an infectious bloodmeal, high levels of JEV (strain CNS138-11) were detected in both the abdomen and head/thorax of all (28/28) surviving females, indicating JEV was successfully acquired and disseminated ( Figure 3). The mean qPCR Ct value for abdomen and head/thorax was 23.58 and 24.19 respectively. Our results also indicate 25/28 (89%) saliva extracts had detectable virus, with a mean qPCR Ct value of 26.93.

### Mitochondrial diversity of *Cx. tritaeniorhynchus* populations

**Figure 4.**
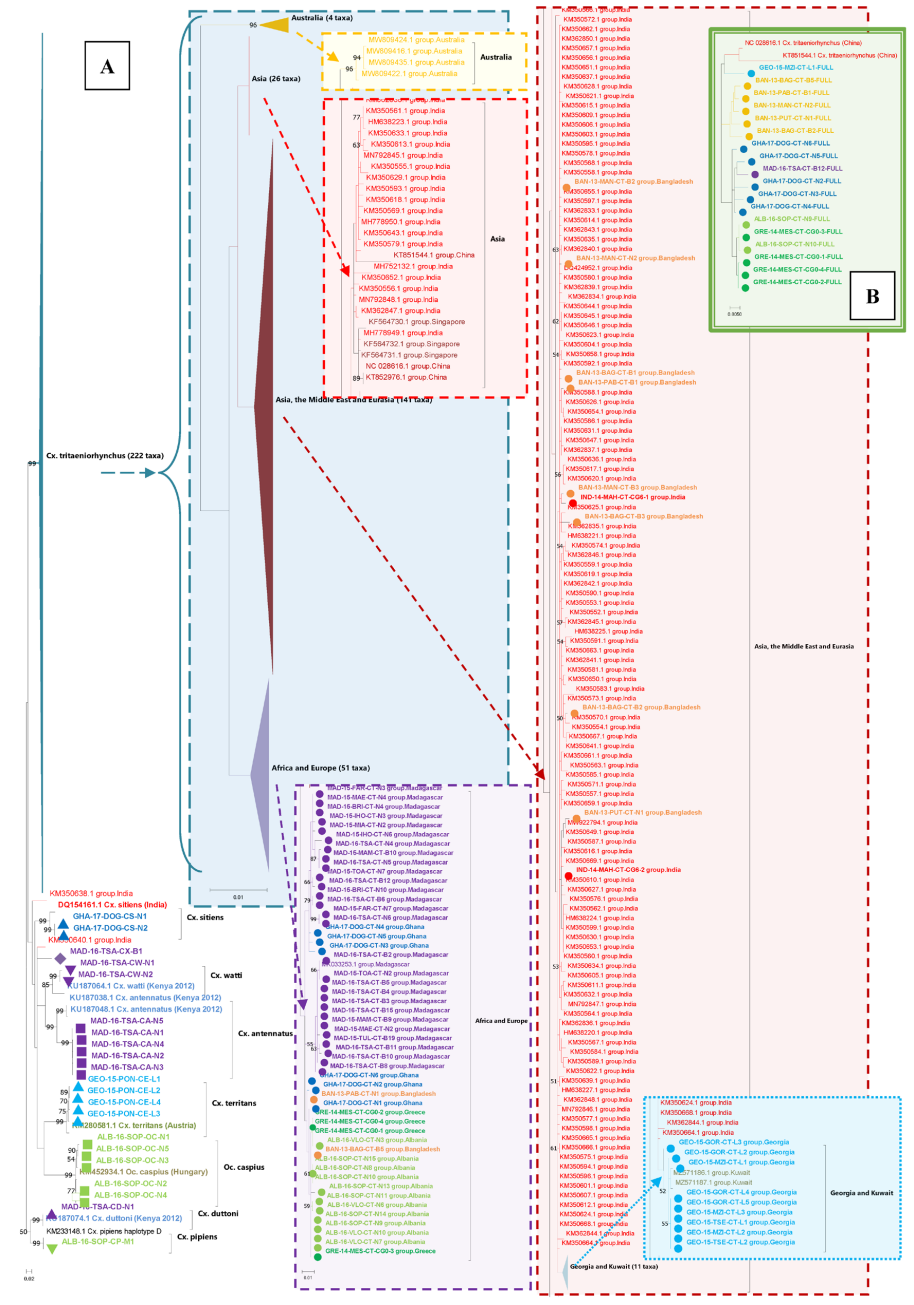
*Cx. tritaeniorhynchus* partial and full *CO1* maximum likelihood phylogenetic trees. ( **A**) Partial *CO1* include concomitant species using GTR model *+G*, *parameter = 1.1140 and [+*I], 61.54% sites. Tree log likelihood = -4937.50, 253 sequences, 686 positions. ( **B**) Full *CO1* gene. Model: T92 + *G*, parameter = 0.0500 and [+ *I*], 57.20% sites. Tree log likelihood = - 2829.05, 20 sequences, 1538 positions.

**Figure 5.**
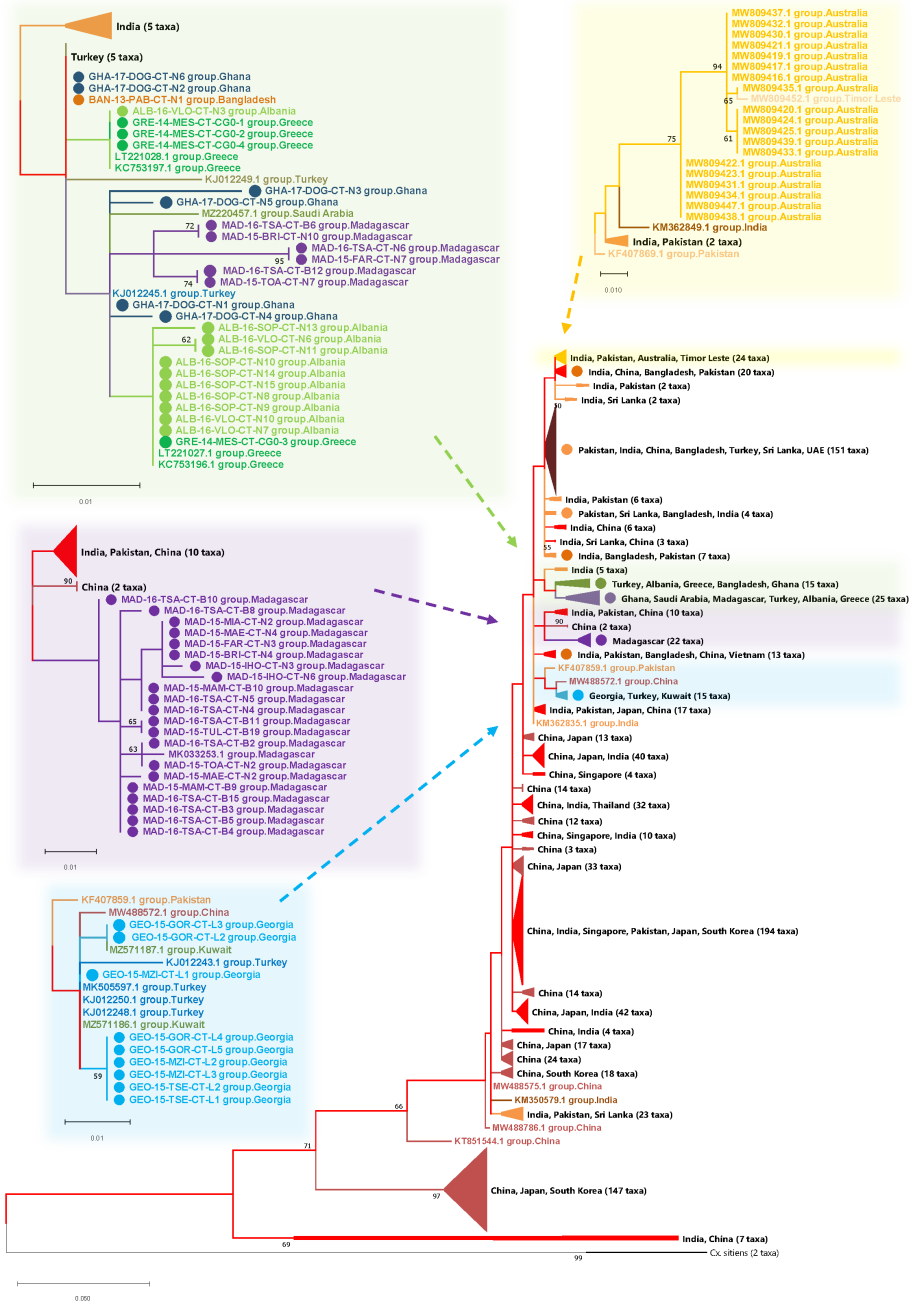
*Cx. tritaeniorhynchus CO1* phylogenetic tree with maximum reference sequences. Partial *CO1* gene, maximum reference sequences. Model: T92 ( Tamura, 1992) + *G*, parameter = 0.3850 and [+ *I*], 47.76% sites. Tree log likelihood = -3611.47. 1009 sequences, 414 positions. Two *Cx. sitiens* sequences included as an outgroup.

Sequences from a total of 69 *Cx. tritaeniorhynchus* specimens, originating from six countries, spread across four continents were generated in this study. Sequence coverage and length was used to generate three *CO1* gene analyses: 1) maximising coverage and comparison of the partial *CO1* gene fragment (primers from ( Kumar *et al*., 2007)) and concomitant species sequences, 253 sequences and 686 nucleotides; ( Figure 4A) 2) maximising the length for comparison of the full *CO1* gene, 20 sequences and 1538 nucleotides; ( Figure 4B) and 3) maximising the number of comparative sequences, with sufficient *CO1* fragment overlap, 1007 sequences and 414 nucleotides which demonstrates complex phylogenetic relationships ( Figure 5). The earliest common ancestors were sequences from India and China and Asian-only clades represent ~70% (673/1007) of sequences. Sequences from Georgia obtained in this study group most closely to sequences from Turkey and Kuwait ( Figure 5 - blue inset sub-tree). The next monophyletic group with more geographically diverse sequences included a group from Madagascar ( Figure 5, purple inset sub-tree). Another clade, developing from a group of Indian sequences, included sequences from Turkey, Ghana, Bangladesh, Albania and Greece ( Figure 5 - green inset sub-tree). Lineages with the greatest extent of genetic distance (using *Cx. sitiens* as an outgroup and Asian ancestral sequences from India and China) were from Australia ( Figure 5 - yellow inset sub-tree), followed by Madagascar and Europe.

### Global mitochondrial diversity metrics

Genetic diversity metrics were generated from 21 countries and 6 regions ( Table 3). When all sequences were compared individually across the 414 nucleotide positions in 1007 sequences, 139 variable sites (S) and 444 haplotypes (h) were identified. The overall haplotype diversity (Hd) was 0.97864, the average number of nucleotide differences (K) was 9.16425, and the nucleotide diversity per site (Pi) was 0.02214. The highest within-country nucleotide diversity per site was seen in South Korea (Pi = 0.03543) and lowest was in Greece (Pi = 0.00129). Asian sequences produced the highest nucleotide diversity per site (Pi = 0.02203), and European sequences had the lowest within-region diversity (Pi = 0.00184).

**Table 3.** *Cx. tritaeniorhynchus* population genetic diversity metrics. Total number of sites: 414. n: Number of samples; S: Number of variable sites; h: Number of haplotypes; Hd: Haplotype diversity; K: Average number of nucleotide differences; Pi: Nucleotide diversity (per site); PiJC: Nucleotide diversity (Jukes-Cantor).

Region	Country	n		S	H	Hd	K	Pi	PiJC
Asia	All (n=11)	909		137	412	0.97487	9.12217	0.02203	0.02265
	Bangladesh	10		13	9	0.97778	2.77778	0.00671	0.00675
	China	608		116	290	0.96684	10.07545	0.02434	0.02509
	Japan	33		40	30	0.99432	12.97159	0.03133	0.03226
	Pakistan	80		30	31	0.84778	2.44399	0.0059	0.00594
	Singapore	6		9	6	1	3.2	0.00773	0.00778
	South Korea	3		22	3	1	14.66667	0.03543	0.03653
	Sri Lanka	7		7	5	0.85714	2	0.00483	0.00486
	Vietnam	1		N/A					
	Thailand	1		N/A					
	Timor-Leste	1		N/A					
Australia	Australia	19		6	4	0.73099	2.33918	0.00565	0.00569
Africa	All (n=2)	34		22	19	0.95544	3.39037	0.00819	0.00825
	Madagascar	28		16	14	0.93651	2.86508	0.00692	0.00697
	Ghana	6		6	5	0.93333	2.2	0.00531	0.00534
Middle East	All (n=3)	4		8	4	1	4.5	0.01087	0.01098
	Kuwait	2		1	2	1	1	0.00242	0.00242
	Saudi Arabia	1		N/A					
	UAE	1		N/A					
Eurasia	All (n=2)	22		13	8	0.85714	3.28571	0.00794	0.008
	Turkey	13		12	6	0.82051	3.35897	0.00811	0.00818
	Georgia	9		2	3	0.55556	0.88889	0.00215	0.00215
Europe	All (n=2)	19		3	4	0.64327	0.76023	0.00184	0.00184
	Albania	11		3	4	0.6	0.69091	0.00167	0.00167
	Greece	8		1	2	0.53571	0.53571	0.00129	0.0013
**Sequence totals**			**1007**	**139**	**444**	**0.97864**	**9.16425**	**0.02214**	**N/A**

### Haplotype networks and pairwise comparison analysis

**Figure 6.**
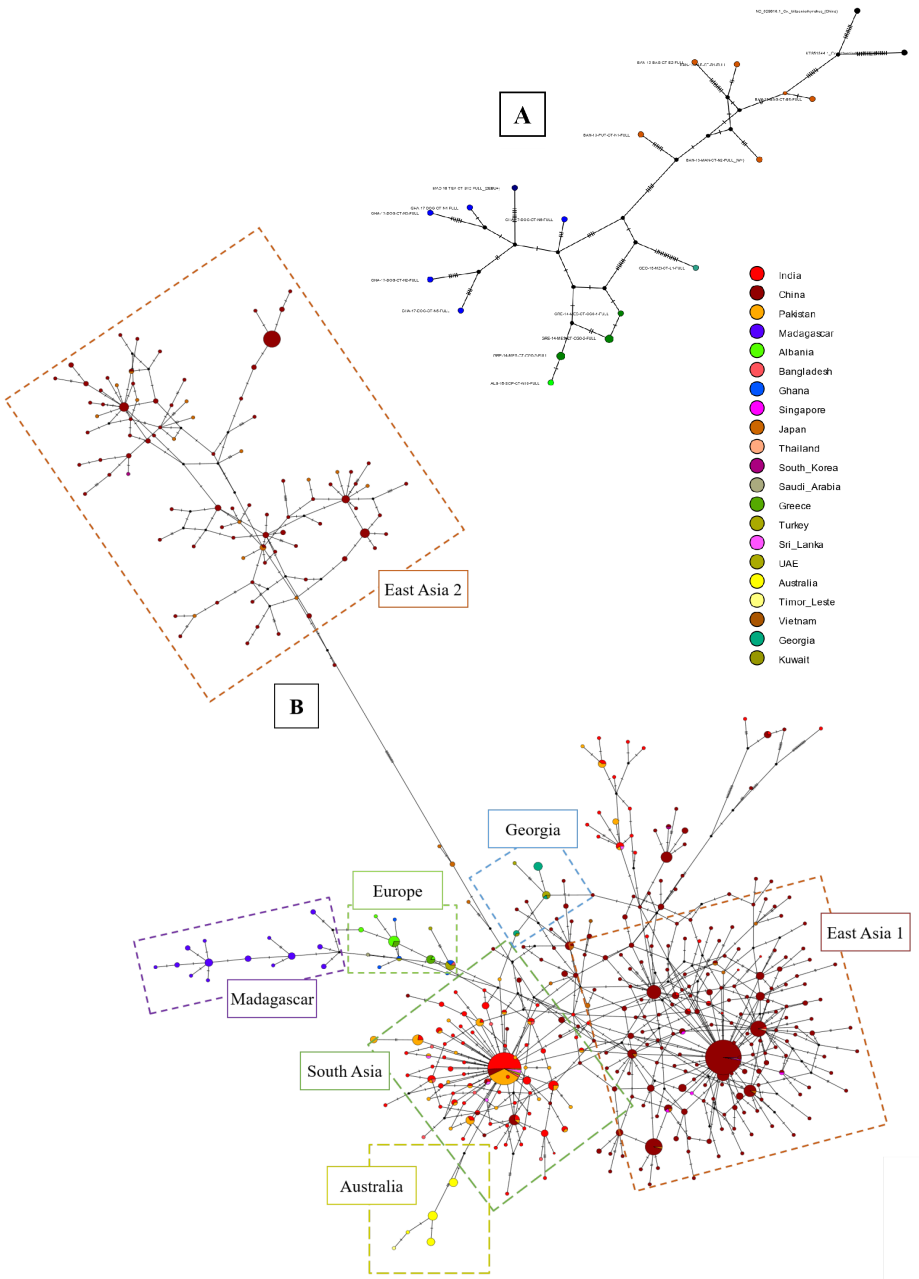
*CO1* haplotype networks for *Cx. tritaeniorhynchus*. ( **A**) Full *CO1* gene haplotype network for *Cx. tritaeniorhynchus* (maximising the length of sequences). ( **B**) Global partial *CO1* haplotype network for *Cx. tritaeniorhynchus* (maximising number of reference sequences). Haplotype networks were constructed using the TCS network method in PopArt ( Leigh & Bryant, 2015) with nodes coloured according to country-of-origin.

**Figure 7.**
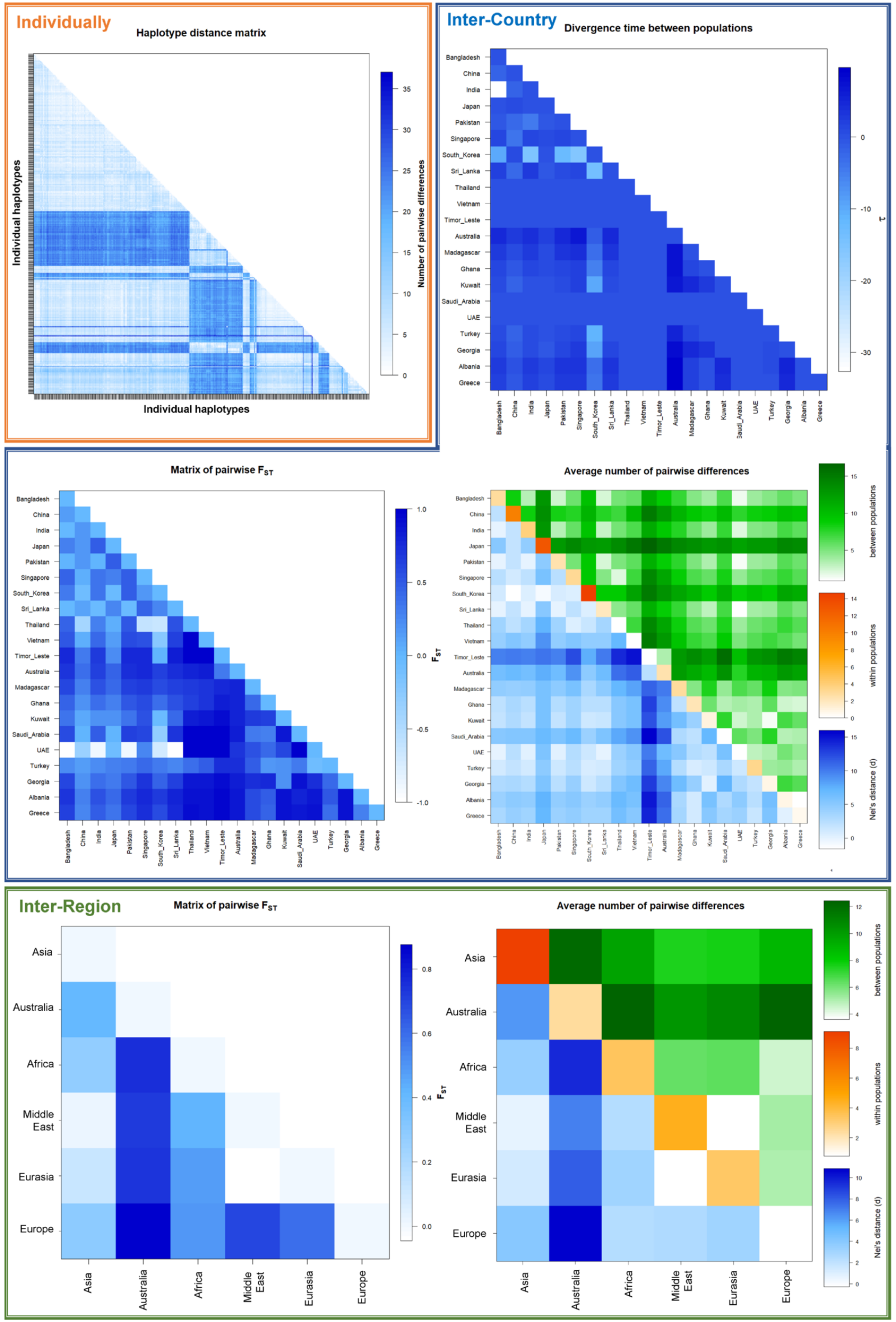
Global genetic diversity Country and Regional populations pairwise comparison heatmaps. *Cx. tritaeniorhynchus* partial *CO1* maximum reference sequences alignment used for haplotype and pairwise analyses within Arlequin software and visualised in R. *F*_ST_ = pairwise fixation index: 0=two populations genetically identical, 1=two populations are genetically different; maximum genetic diversity between two populations.

*CO1* haplotype networks using full-length *CO1* gene sequences (20 sequences, 1500 positions) ( Figure 6A) suggested a linear pattern with sequences from Asia clustering separately to sequences from Eurasia, Europe and Africa (the latter being most divergent from the Asia haplogroup). Partial *CO1* gene sequences (1007 sequences, 414 positions) ( Figure 6B) produced a more complex haplotype network with one group from South Asia and two groups from East Asia. Haplotypes found in Australia and Timor-Leste appear to be branching from the large South Asian cluster. The haplotypes from Georgia appeared to branch from haplotypes present in Turkey and the Kuwait, sitting between the two large South, and East Asian foundational haplogroups. In a separate cluster, haplotypes from Greece and Albania branched off from the main South Asian haplogroup, linked alongside some haplotypes present in Turkey and Ghana. The haplotype present in Saudi Arabia also originated from this branch, and the haplotypes present in Madagascar then extended and diverged further from the end of this branch. Pairwise comparison analysis heatmaps for differences in genetic diversity within and between populations ( Figure 7) revealed sequences from Australia as having the greatest difference to other countries/regions, and sequences from East Asia having the greatest intra-group differences. The matrix of pairwise fixation index (F _ST_) indicates high genetic differentiation between populations in different countries and regions, particularly for the Australian, as well as the African and European groups. The divergence time between populations is also relatively lower for these populations.

### Bacteria identified using microbiome analysis

We compared the composition and diversity of microbes present between collection locations or countries, physiological states (blood-fed or non-blood-fed), or between *Cx. tritaeniorhynchus* and concomitant mosquito species ( Table 2). Species of potential relevance to mosquito biocontrol were detected including *Wolbachia*, *Asaia*, *Serratia*, *Pseudomonas* and *Apibacter* but presence and abundance levels were variable across individuals and populations. From 57 Madagascar samples, 34 had *Apibacter* (0.05–99.87% relative abundance), seven had *Pseudomonas* (1.0–29.92%), three had *Asaia* (1.30–34.06%) and one had *Serratia* (20.38%). Pathogenic bacteria were also detected including *Escherichia shigella*, *Vibrio cholerae* and species within the *Bartonella*, *Anaplasma*, *Rickettsia*, *Mycoplasma*, *Enterobacter*, *Helicobacter* and *Providencia* genera. *Bartonella* was found in an Albania sample at a relative abundance of 27.45%. *Escherichia shigella* was found in two Albanian samples (5.81% and 0.34% relative abundance) with this second sample also containing *Vibrio cholerae* at 6.28%. In Madagascar, *Escherichia shigella* was identified in five specimens with abundance ranging from 0.41–30.00%, *Bartonella* in one specimen with 45.32% abundance and this specimen also had *Pseudomonas* at 8.63% abundance. *Anaplasma* was also found in Madagascar samples with relative abundance ranging from 5.02–73.71%. Division down to taxonomic level 7 showed these ASVs were identified as *Anaplasma marginale*, *Anaplasma platys* and the rest classified within the *Anaplasma* genus. *Mycoplasma* was also identified in seven individuals (0.33–21.61%) and *Escherichia shigella* in six individuals from Madagascar (0.03–1.91%).

### Microbiome diversity analysis

Concomitant species comparisons were possible for mosquitoes collected from Fier in Albania and Tsaramandroso in Madagascar. For Fier, gDNA samples from whole, non-blood-fed *Cx. tritaeniorhynchus* (n=16), *Cx. pipiens* (n=16) and *Oc. caspius* (n=16) specimens demonstrated variation in microbial composition. Alpha diversity within each species group showed no significant differences, however, beta diversity analysis showed clear differences between the species (Bray-Curtis p=0.001 and Weighted-Unifrac p=0.005). ANCOM identified *Wolbachia* as the only significant differentially abundant feature between the groups (W=265) due to high abundance in *Cx. pipiens*. From Tsaramandroso, cDNA extracted from abdomens of non-blood-fed female *Cx. tritaeniorhynchus* (n=12) and *Cx. antennatus* (n=14) demonstrated no significant difference in alpha or beta diversity and no significant differentially abundant taxa in ANCOM analysis. Comparisons were also made from abdomens of non-blood-fed and blood-fed female specimens collected from Fier (non-blood-fed n=15, blood-fed n=12) and Tsaramandroso (non-blood-fed n=12, blood-fed n=15). For both countries, alpha- and beta-diversity and ANCOM highlighted no differences which were statistically significant.

### Microbiome variation of intra-country *Cx. tritaeniorhynchus* populations

For intra-country comparisons specimens from two sites 35km apart in Bangladesh were compared: Paba (n=10) and Bagmara (n=7). No statistically significant differences were found through alpha- or beta-diversity, or ANCOM analysis. Within Albania, cDNA from non-blood-fed female abdomens also collected 35 km apart from Fier (n=15) and Vlore (n=10) were compared. The differences between individuals within these groups were found to be significant (Faith’s phylogenetic diversity metric, p=0.0087), as well as between the groups (Unweighted-Unifrac, p=0.016) and *Enterobacteriaceae* was found to be significantly differentially abundant (ANCOM, W=156), with a higher abundance in Vlore than in Fier. For Madagascar, the microbiome data was generated from cDNA extracted from whole non-blood-fed females from 6 sites; Brickaville (n=10), Farafangana (n=9), Ihosy (n=10), Maevatanana (n=9), Miandrivazo (n=10) and Toamasina (n=10). Alpha diversity did not highlight any significant difference between individuals within groups, but beta-diversity showed a difference between the locations (overall Weighted-Unifrac, p=0.003), with significance between Brickaville-Miandrivazo (p=0.012), Farafangana-Miandrivazo (p=0.012), Ihosy-Miandrivazo (p=0.001) and Maevatanana-Miandrivazo (p=0.004). ANCOM, however, found no significant differentially expressed taxa.

### Inter-country microbiome comparisons

Samples from Bangladesh and Albania (gDNA, whole-body, non-blood-fed) were compared and alpha-diversity demonstrated no significant difference between individuals within each group, whereas beta-diversity highlighted differences between each country, with Bray-Curtis (p=0.001) and Unweighted-Unifrac (p=0.001). ANCOM analysis showed there were several differentially abundant taxa between the groups from Bangladesh and Albania. The three taxa which were most statistically significant were two *Erwinia* species (W=235 and W=224) and *Asaia* (W=219), with their abundance in Albania much greater than in Bangladesh.

For comparisons between Albania and Madagascar, one comparison was made between non-blood-fed and the other between blood-fed *Cx. tritaeniorhynchus* from the two countries (cDNA, abdomen samples). For non-blood fed, none of the alpha- or beta-diversity indexes showed significant results but ANCOM highlighted *Anaerobacillus* as a significant differentially abundant taxa (W=260), with higher abundance in Madagascar than Albania. This genus was present in 9/12 samples from Tsaramandroso, Madagascar (relative abundance range of <1% to 6.08%), present in 4/10 samples from Vlore, Albania, (<1% to 1.99%) and in 0/15 samples from Fier (Sop), Albania. For the comparison between blood-fed females across the two countries, alpha-diversity showed no significant difference between the individuals in each group; from Fier (Sop), Albania (n=12) and Tsaramandroso, Madagascar (n=15). The Bray-Curtis beta-diversity metric showed a significant difference between the groups from each country (p=0.003) and ANCOM highlighted that reads classified in the *Bacillus* genus were significantly differentially abundant (W=266), with a relatively high abundance in samples from Madagascar, and absent from the blood-fed mosquitoes from Albania.

### Detection and phylogeny of *Wolbachia* bacteria strains

**Figure 8.**
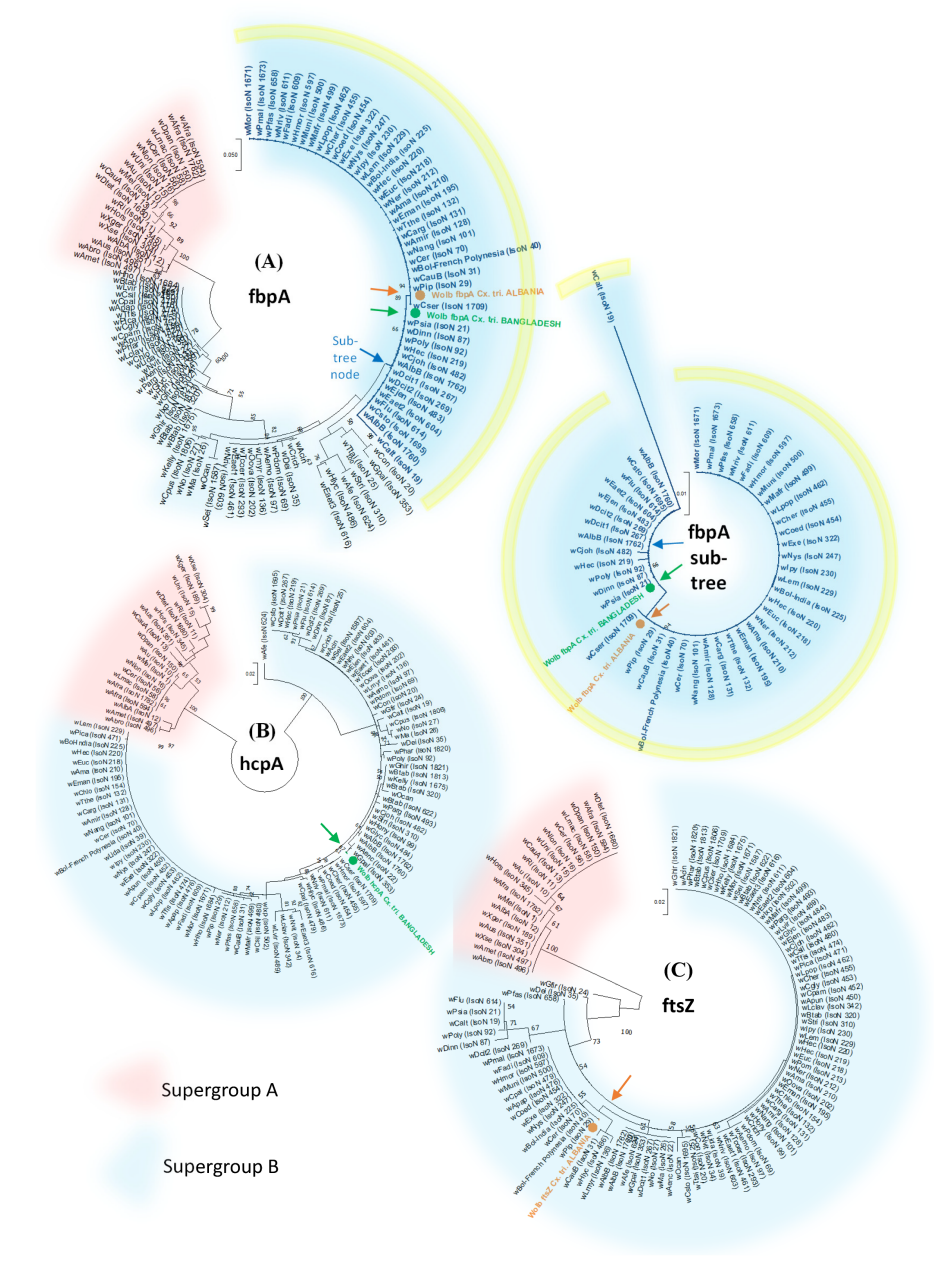
*Wolbachia* MLST gene phylogenetic trees. The T92 model ( Tamura, 1992) was used for all. *Wolbachia* Supergroups A and B are highlighted in red and blue respectively. The sequences obtained from *Cx. tritaeniorhynchus* from Albania and Bangladesh are shown in orange and green respectively. ( **A**) *fbpA* gene locus. Tree log likelihood = -1947.41 (+ *G*, parameter = 0.2126). The analysis involved 113 nucleotide sequences and 429 positions. An *fbpA* sub-tree is also shown separately (yellow border) for more detailed visualisation of the *Wolbachia fbpA* sequences obtained from *Cx. tritaeniorhynchus*. ( **B**) *hcpA* gene locus. Log likelihood = -1825.87. (+ *G*, parameter = 0.3916). 111 nucleotide sequences and 444 positions. ( **C**) Partial *ftsZ* locus. Log likelihood = -535.33 (+ *G*, parameter = 0.2600). 113 nucleotide sequences and 189 positions.

Taxonomic abundance analysis from microbiome analysis did show evidence for the presence of *Wolbachia* in *Cx. tritaeniorhynchus* samples from Bangladesh, Albania and Madagascar. One sample from Bagmara, Bangladesh exhibited a relative abundance of *Wolbachia* comprising 39.77% of the total microbial composition, and two further specimens from the same location had 5.94% and 4.72% relative abundance. A blood-fed specimen collected in Fier (Sop), Albania, had a *Wolbachia* relative abundance of 22.50%. Three non-blood-fed samples from Albania, collected in Fier (Sop) and Vlore, also showed the presence of *Wolbachia* with relative abundances of 5.88%, 1.11% (Sop) and 1.32% (Vlore). Concomitant Albanian *Cx. pipiens* mosquitoes (a species known to be naturally infected with the wPip strain of *Wolbachia*) were shown to have variable *Wolbachia* relative abundances, ranging from 0–38.24%. Confirmation of *Wolbachia*-specific 16S rRNA amplification was possible for some samples from Albania and Bangladesh ( Table 4). Further analysis using *Wolbachia* MLST showed a variable pattern of amplification and sequencing but partial MLST profiles were obtained for samples from Albania and Bangladesh ( Table 5). Partial MLST allelic profiles and phylogenetic analysis ( Figure 8) indicated that these *Wolbachia* strains were different from one another but both placed within Supergroup B. Using the *Wolbachia* fbpA locus, the two strain do appear closely related ( Figure 8).

**Table 4.** *Wolbachia* positive *Cx. tritaeniorhynchus* samples. *Wolbachia* testing success through *16S* microbiome analysis, *Wolbachia*-specific *16S* qPCR, or *Wolbachia* MLST gene PCRs. + denotes successful amplification/detection, - denotes *Wolbachia* not detected through this method, *denotes successful sequencing. NBFF – Non-blood-fed female, BFF – Blood-fed female.

Location (year)	Sample ID	16S	16S qPCR	gatB	coxA	fpbA	ftsZ	hcpA
		V3-V4						
Bagmara, Rajshahi, Bangladesh (2013)	Bang T6 109 1 (AB2) NBFF	+	+					
	Bang T6 109 2 (AB3) NBFF	*-*	*+*					
	Bang T6 109 3 (AB4) NBFF	*+*	*+*					
	Bang T6 110 1 (AB5) NBFF	*+*	*-*					
Manda, Naogaon, Bangladesh (2013)	Bang T6 196 2 BFF	-	+	+	+	+ *	+	+ *
	BG0 T6 194 1 NBFF		+		+	+		
	BG0 T6 194 1 NBFF		*+*					
	BG0 T6 194 3 BFF		*+*					
Shengjin, Lezhe, Albania (2015)	AG0 T222 BFF1		+	-	+	+ *	+ *	+
	AG0 T222 BFF2		*+*					
	AG0 T222 BFF3		*+*					
Sop, Fier, Albania (2015)	AG0 T201BFF		*+*					
Sop, Fier, Albania (2017)	ALB-17-SOP-CT-N12-WD (gDNA) BBF		*+*					

**Table 5.** *Wolbachia* partial MLST gene allelic profiles for resident strains in *Cx. tritaeniorhynchus* populations. “CM” = Allele number of the closest allelic match, with the number of nucleotide differences in brackets. “EM” = Exact match on that locus to the allele number provided. “SG” = Super group to which isolates with that allele at that locus belong. *denotes where the query sequence was truncated, therefore the full locus wasn’t available for comparison. “-” denotes where sequencing was attempted from PCR products but the sequence data quality wasn’t sufficient for analysis. “NS” denotes where no clear PCR amplified product was obtained and therefore sequencing was not attempted.

Country and sample ID	*gatB*	*coxA*	*hcpA*	*ftsZ*	*fbpA*
Bangladesh – BG0 T6 196 2 F9	-	-	CM 12 (1)	-	**EM 27**
			SG B		SG B
Albania – AG0 T222 BFF1 E9	NS	-	-	CM 280 * (0)	**EM 4**
				SG B	SG B

## Discussion

Despite the presence of *Cx. tritaeniorhynchus* first being reported in Albania in 1960 ( Danielovi & Adhami, 1960), further published European occurrence reports were scarce until the 2000s when this species was recorded in Greece ( Lytra & Emmanouel, 2014a, Lytra & Emmanouel, 2014b; Patsoula *et al*., 2017; Samanidou & Harbach, 2003). Recent extensive entomological surveys carried out in Albania (including this study) have identified *Cx. tritaeniorhynchus* within multiple areas for the first time ( Alves *et al*., 2014; Gugushvili, 2002; Lessard *et al*., 2021; Samanidou & Harbach, 2003), highlighting a trend towards expansion of the known geographical range. Entomological surveillance in Europe has also identified other invasive mosquito vector species such as *Aedes albopictus*, highlighting the risk of exotic vector species becoming established ( Medlock *et al*., 2012). Concurrently, there has been an increase in outbreaks and circulation of mosquito-borne arboviruses such as WNV in Europe ( Bakonyi *et al*., 2013; Calzolari, 2016; Engler *et al*., 2013). To our knowledge, this study is the first to assess JEV vector competence in a European population of *Cx. tritaeniorhynchus* and our results emphasise the possibility of future introductions and JEV epidemics outside of Asia. Previous detection of JEV RNA in mosquitoes and birds in Italy further reinforces this point ( Platonov *et al*., 2012; Ravanini *et al*., 2012).

In our study, a lower number of saliva samples (compared to mosquito body parts) had detectable virus after the 14-day incubation which might be expected, as the excretion of virus in the saliva is the final process in the infection pathway, following after viral acquisition and dissemination. Caution must be taken in over-extrapolating these results given 1) we were unable to generate comparative vector competence data between geographically dispersed populations due to colonisation difficulties and 2) direct extrapolation of laboratory vector competence to wild populations is likely to be imprecise given the complexity of transmission dynamics in wild populations. Comparisons to previous studies are also problematic due to variation in infection and detection methods. For example, a study in Korea resulted in 33–67% JEV transmission (via capillary tube saliva collection, or onward infection of chickens) ( Turell *et al*., 2006) and in India, using ELISA in whole bodies, variable infection rates were reported, from 0–48% ( Philip Samuel *et al*., 2010). The relatively lower infection rates seen in the latter study may be, at least in part, a result of reduced sensitivity of ELISA for virus detection or differences in JEV infectious doses.

Our study should enable a more accurate taxonomic classification of *Cx. tritaeniorhynchus* – particularly important as hybridisation within species complexes (e.g. *Cx. pipiens*) can influence arbovirus transmission ( Shaikevich *et al*., 2016). The mitochondrial *CO1* gene has been most frequently used ( Ashfaq *et al*., 2014; Karthika *et al*., 2018; Kumar *et al*., 2007; Li *et al*., 2020; Rajavel *et al*., 2015; Xie *et al*., 2021) allowing species discrimination and investigation of maternal inheritance patterns ( Cywinska *et al*., 2006; Hebert *et al*., 2003; Karthika *et al*., 2018). Our genetic diversity metrics quantified the genetic distances and divergence within population groups, identifying 444 haplotypes and 139 variable sites, with a haplotype diversity of 0.97864 and a nucleotide diversity per site of 0.02214. To our knowledge this is the first published study examining such a geographically diverse mitochondrial dataset – previous studies have identified 28 ( Karthika *et al*., 2018) and 303 ( Xie *et al*., 2021) haplotypes, with the latter finding a Hd of 0.97 and Pi of 0.02434 which is comparable to our study. Analysis of regional population groups identified 412 haplotypes in Asia, four in Australia, 19 in Africa, four in the Middle East, eight in Eurasia and four in Europe. The only previous study for European *Cx. tritaeniorhynchus* found two haplotypes within the same population, collected in a single rice field in western Greece ( Lytra & Emmanouel, 2014b). Our samples from the same location identified two haplotypes which were also present in Albania, and a further two haplotypes were found in Albania only.

The haplotype network and pairwise comparison analysis also indicated that (as expected) geographical location influences mitochondrial diversity. For example, there was a distinct grouping of 14 haplotypes in Madagascar, none of which were found in any other countries or regions. The greater genetic distances of some groups, such as Australia, Madagascar, and Europe, is logical but are genetic bottlenecks or selection pressures occurring during adaptation to new environments? Maternal lineages can provide insight into possible movement patterns and although *Cx. tritaeniorhynchus* are estimated to have an average flight distance of just under 70 metres, some studies have found that during long-distance wind-assisted dispersal, they can migrate 200–500 kilometres ( Verdonschot & Besse-Lototskaya, 2014). As adults overwinter, this species may use a combination of long-distance migration and hibernation *in situ,* as strategies to survive unfavourable conditions in temperate regions ( Min & Xue, 1996). The ability to disperse over such long distances and adapt to variable conditions is likely to provide more opportunities for range expansion and to increase gene exchange among different populations ( Xie *et al*., 2021).

Our microbiome analysis revealed some bacteria such as *Asaia* that was present in all populations and differentially abundant in Albania when compared to Bangladesh. However, as *Asaia* can be environmentally acquired ( Favia *et al*., 2007), it may depend on differing exposures in local habitats, rather than a country-wide distinction. In Albania, *Cx. pipiens* had a greater abundance of *Wolbachia* (ANCOM, W=265) but a lower abundance of *Asaia* than the other two species. Although not statistically significant and some individuals had both *Wolbachia* and *Asaia*, further studies should be undertaken to determine any antagonistic associations as seen in mosquito lab colonies ( Hughes *et al*., 2014; Rossi *et al*., 2015). *Apibacter* was detected in Madagascar samples (both at high prevalence and for some indiviudals high relative abundance) and in individuals from Bangladesh and Albania. *Apibacter* have been relatively recently isolated and classified in 2016 from various bee species ( Kwong & Moran, 2016; Praet *et al*., 2016), as well as being reported in house flies ( Park *et al*., 2019) and *Cx. fuscocephala* from Thailand ( Thongsripong *et al*., 2021). These bacteria are thought to be beneficial endosymbionts with characteristics of adaptation to the gut environment and a degree of host-specificity ( Kwong *et al*., 2018). *Apibacter* may also confer a degree of protection against pathogens with a recent study finding an association between *Apibacter* in bees and decreased infection by a trypanosomatid gut parasite *Crithidia bombi* ( Kwong *et al*., 2018; Mockler *et al*., 2018).

Several genera which contain pathogenic species including *Anaplasma, Rickettsia, Bartonella, Vibrio, Helicobacter, Providencia, Mycoplasma* and *Escherichia* ( Christou, 2011) were identified but it was not possible to classify the ASVs beyond genus level to determine whether they were pathogenic species. *Escherichia shigella* and *Vibrio cholerae* were identified and can cause dysentery and severe cholera respectively. These pathogenic bacteria may have been present in local aquatic environments and environmentally acquired. However, detection does not imply *Cx. tritaeniorhynchus* has the capacity for onward transmission, although it may theoretically be possible to mechanically disperse bacteria between water sources. *Anaplasma marginale* and *Anaplasma platys,* which can cause anaplasmosis in cattle and dogs respectively, are vector-borne pathogens, although they are mainly thought to be transmitted by ticks. A recent study in China found a wide range of *Rickettsiales*, including *Anaplasma* spp., in mosquito species, including *Cx. tritaeniorhynchus* ( Guo *et al*., 2016). Phylogenetic analysis suggested a potential role for mosquitoes in vector-borne transmission of *Anaplasma marginale*, with other *Anaplasma* species suggested to be vertically transmitted ( Guo *et al*., 2016). Finding these bacteria at relatively high abundance in blood-fed mosquitoes in our study, and not in the matched non-blood-fed mosquitoes may suggest these bacteria were present in the blood meals and not disseminated infections. Even without vectorial capacity, mechanical transmission during blood-feeding may be possible and the high abundance in blood-fed-females would suggest a relatively frequent exposure to *Anaplasma*, particularly *Anaplasma marginale,* from feeding on cattle. Bacteria from the *Bartonella* genus are also vector-borne, with ticks, fleas, lice and sandflies implicated as vectors ( Billeter *et al*., 2008; Jacomo *et al*., 2002). *Bartonella* species can infect humans and animals causing bartonellosis. Although *Bartonella* was only identified in a few *Cx. tritaeniorhynchus* individuals from each country, when present it was highly abundant. *Bartonella* was found dominating the microbiome in 4/13 concomitant *Cx. antennatus* specimens in Madagascar despite none found in the matched group of *Cx. tritaeniorhynchus* from the same location.

Our microbiome analysis identified the presence of *Wolbachia* in populations with variable levels of relative abundance (0–40%) suggesting the likely presence of multiple strains at low prevalence spread across several continents. Phylogenetic analysis of the *Wolbachia 16S* and MLST gene loci confirms the strains from individuals in Albania and Bangladesh are placed within Supergroup B. Although the strains do differ from one another where comparison was possible on the *Wolbachia fbpA* locus, they appear to be closely related. Some previous studies have not identified *Wolbachia* in *Cx. tritaeniorhynchus* ( Kittayapong *et al*., 2000; Nugapola *et al*., 2017; Ravikumar *et al*., 2010; Tsai *et al*., 2004), however, a study from Thailand ( Wiwatanaratanabutr, 2013) and recently from Singapore ( Ding *et al*., 2020), reported *Wolbachia* in small numbers of individual mosquitoes. *Cx. tritaeniorhynchus* is implicated as a vector of *Dirofilaria immitis*, a filarial nematode with an obligatory symbiotic relationship with *Wolbachia*, requiring its presence for survival. However, phylogenetic analysis carried out in our study indicates the *Wolbachia* strains are not likely to result from filarial infections given the placement within Supergroup B ( Dyab *et al*., 2016). It remains to be determined whether these *Wolbachia* strains influence reproductive success through the cytoplasmic incompatibility phenotype, are vertically transmitted with high rates of maternal transmission, or impact vectorial capacity. Low infection rates may suggest they do not possess the beneficial phenotypic characteristics most useful for mosquito biocontrol and may not inhibit arboviruses as seen in some other studies on native strains ( Tsai *et al*., 2006). However, low-level natural *Wolbachia* strains in *Cx. tritaeniorhynchus* populations are unlikely to be prohibitive to the development of *Wolbachia*-based biocontrol strategies, such as through transinfection of non-native strains. Further analysis of larger sample numbers from diverse geographical areas is needed including non-PCR based methods such as microscopy to visualise bacteria in mosquito tissues ( Walker *et al*., 2021).

## Conclusions

Our study provides evidence that a European population of *Cx. tritaeniorhynchus* is a competent vector of JEV and shows that a high degree of mitochondrial and microbial diversity is present across geographically dispersed populations of this species. Future vector competence experiments should incorporate factors, such as temperature, to more closely mimic the current and potential future environmental niches inhabited by this vector. Establishing whether geographically diverse populations of *Cx. tritaeniorhynchus* are competent vectors for other medically important arboviruses such as WNV and RVFV is also a priority as exemplified by it's involvement in the first incursion of RVFV outside of Africa. This study also provides evidence for the presence of diverse bacteria, including those considered candidates for biocontrol, and pathogenic bacteria. Further studies should be undertaken to determine if *Wolbachia* strains naturally reside within *Cx. tritaeniorhynchus* populations. Increased surveillance and novel control strategies for *Cx. tritaeniorhynchus* will be important with climate change increasing the range of this invasive species which can transmit multiple arboviruses of concern to public health.

## Data Availability

Open Science Framework: Microbiome data associated with. Jeffries *et al*. Mitchondrial and microbial diversity of the invasive mosquito vector species Culex tritaeniorhynchus across its extensive inter-continental geographic range. https://doi.org/10.17605/OSF.IO/DJ7WA ( Jeffries & Walker, 2023) This project contains the raw 16S sequencing reads in FASTA format. Data are available under the terms of the Creative Commons Zero “No rights reserved” data waiver (CC0 1.0 Public domain dedication).
