# Supplementary material for: Mitochondrial and microbial diversity of the invasive mosquito vector species Culex tritaeniorhynchus across its extensive inter-continental geographic range
Source: Wellcome Open Res. 2024 Jan 12;9:18. [Version 1] doi: 10.12688/wellcomeopenres.20761.1 (PMC11128058; doi:10.12688/wellcomeopenres.20761.1)
Supplement: Peer review history

# Reviewer report: Reviewer response for version 1

**DOI:** 10.21956/wellcomeopenres.22976.r76780

**Published:** 2024-05-25

**Authors:** Yasmina Martínez Barciela

**Affiliations:** 1 Universidade de Vigo, Vigo, Spain

The work is focused on the study of three aspects related to *Culex tritaeniorhynchus*: the vector competence of Greek populations to transmit Japanese encephalitis virus (JEV) under laboratory conditions (i), the phylogenetic relationship between populations from different continents by mitochondrial analysis using the *cytochrome oxidase subunit 1 (CO1)* gene (ii), and the microbial diversity (such as *Wolbachia*) naturally present in the different populations by bacterial 16S rRNA gene amplicon sequencing and multi-locus sequence typing (MLST).

 The results obtained are novel and of interest not only in phylogenetic terms, but also regarding vector surveillance and environmental health. The manuscript is well developed in easily understandable English.

 Regarding the structure, the different sections are discussed:

 Abstract. It is well structured. A spelling mistake is detected in the form of the verb “to be” in the first line of the results.

 Introduction. Appropriate length and content. It is recommended not to abbreviate Rift Valley fever virus (RVFV) and West Nile virus (WNV) the first time they are used. When the species *Culex tritaeniorhynchus* is mentioned at the beginning of a sentence, the criteria should be unified: either with or without the abbreviation for the genus ( *Cx.*) (it is recommended not to abbreviate it in these cases).

 Methods. Appropriate structure and explanations apparently sufficient to be able to replicate the method of molecular analysis. However, the information on the capture of adult specimens in the field is not sufficiently well specified (what type of traps are used in each case?). Likewise, it would be useful to add the sampling period including the months of the year and not only the years of sampling.

 Results: Well structured and adequately explained for their understanding.

 Discussion. Well supported by the bibliography and easy to read. When mentioning the pathogenic species found in *Culex tritaeniorhynchus*, it would be interesting to clarify in which populations (countries) each pathogens were found (this information is not clarified in all cases).

 Conclusions. Appropriate length and content.

 References. Adequate length and updated content. No errors have been detected.

 Regarding the quality of this review, it is important to point out that it may be necessary to have experts in phylogenetics, molecular analysis and statistics to make a more appropriate assessment of the study.

Is the work clearly and accurately presented and does it cite the current literature?

Yes

If applicable, is the statistical analysis and its interpretation appropriate?

I cannot comment. A qualified statistician is required.

Are all the source data underlying the results available to ensure full reproducibility?

Yes

Is the study design appropriate and is the work technically sound?

Yes

Are the conclusions drawn adequately supported by the results?

Yes

Are sufficient details of methods and analysis provided to allow replication by others?

Partly

Reviewer Expertise:

Entomology, vectorial ecology, environmental health

I confirm that I have read this submission and believe that I have an appropriate level of expertise to confirm that it is of an acceptable scientific standard.

# Reviewer report: Reviewer response for version 1

**DOI:** 10.21956/wellcomeopenres.22976.r76774

**Published:** 2024-05-03

**Authors:** Evangelina Muttis

**Affiliations:** 1 Investigadora Asistente en CEPAVE, Buenos Aires, Argentina

The study comprises three distinct analyses of *Culex tritaeniorhynchus*, the primary vector of Japanese Encephalitis Virus (JEV), across six countries and four continents: genetic divergence of COI sequences, bacterial diversity determined by amplification of the bacterial 16S rRNA gene, and studies of vector competence within the mosquito population for JEV transmission.

 Firstly, significant genetic divergence of the COI gene among populations was demonstrated. The provided information serves as a dataset for future research endeavors in vector biology or taxonomic studies.

 Secondly, the investigation into microorganisms associated with this mosquito vector holds relevance due to potential interactions with arboviruses. Therefore, data on certain populations naturally infected with Wolbachia represent an initial stride towards considering vector control strategies.

 Lastly, studies on vector competence revealed that Greek *Cx. tritaeniorhynchus* mosquitoes are proficient in transmitting JEV, at least under laboratory conditions, exhibiting a high infection rate.

 While the content of this article remains pertinent, several inquiries emerge from it. For instance, are there correlations among the factors under study? Is microbial diversity associated with mitochondrial diversity? Can JEV outbreak zones be compared with the factors studied? In my perspective, the various topics addressed should be interconnected in some manner.

Is the work clearly and accurately presented and does it cite the current literature?

Yes

If applicable, is the statistical analysis and its interpretation appropriate?

I cannot comment. A qualified statistician is required.

Are all the source data underlying the results available to ensure full reproducibility?

Yes

Is the study design appropriate and is the work technically sound?

Yes

Are the conclusions drawn adequately supported by the results?

Yes

Are sufficient details of methods and analysis provided to allow replication by others?

Yes

Reviewer Expertise:

Medical entomology

I confirm that I have read this submission and believe that I have an appropriate level of expertise to confirm that it is of an acceptable scientific standard.

# Reviewer report: Reviewer response for version 1

**DOI:** 10.21956/wellcomeopenres.22976.r76789

**Published:** 2024-04-15

**Authors:** Piotr Lukasik, Diana Rojas-Guerrero

**Affiliations:** 1 Jagiellonian University, Kraków, Poland

The manuscript “Mitochondrial and microbial diversity of the invasive mosquito vector species *Culex tritaeniorhynchus* across its extensive inter-continental geographic range” addresses a set of significant questions about the biology of a broadly distributed mosquito species recently introduced to Europe. The authors ask about the species’ ability to transmit the Japanese encephalitis virus, its mitochondrial diversity, how it is distributed across the species’ range, and its microbiota, with emphasis on Wolbachia. They address the first question experimentally and others using different types of sequencing.

 I consider the results largely reliable, and I do think that the study advances our understanding of a significant species. However, I do have several major concerns.

My first major issue is the strong overall imbalance among article sections. **The results for the first section** (experiment with viruses) are shown using a single main-text figure, which I think is about the right amount of space. In turn, **the second portion of results **- the comparison of 69 newly generated COI sequences against resources from Genbank - is shown across six main-text display items (one table and five figures). In my view, this is excessive. There is lots of redundancy among figures, and I feel that because of this, the overall “take home” message of these analyses is obscured. I would strongly recommend combining the most important conclusions of phylogenetic work into one main-text figure (and definitely not more than 2), and moving the remaining display items to supplement.

 Further, **the results for the fourth section** - the summary of amplification efforts and analysis of ca. six short Wolbachia sequences in comparison to resources from public databases - are shown using three main-text display items (two tables and one figure) - again, more than necessary, in my view. I see no justification for Tables 4 & 5: results shown in each of them can be fully described in two-three sentences max.

 Strikingly, **for the third section, **comprising the most complex data - the analysis of microbiome data for >100 specimens - the authors chose not to provide any display items at all. [Table 2 lists populations used for the microbiome survey, but not the results]. I was confused about the author’s decision to only verbally summarize some of these results. I strongly feel that some display items are needed. The authors should visualize the distribution of microbial clades (genera?) across samples - in my view, a heat map would be most effective means. They should consider plotting the results of some of their microbiota comparisons within or among populations, using PCoA plots or similar means.

Another major problem is the availability of the data. Results of viral screens are shown in the figure, but the underlying data are not provided. Also, I do not believe that the COI or Wolbachia sequences, alignments, or trees were made available publicly: at least, accession numbers are not shown. The results of microbiome analyses are not shown at all: the authors need to provide the analyses underlying conclusions as supplementary tables. While they posted their raw amplicon data in OSF.IO database, I could not see metadata for these fastq.gz files - there is no information on  which file corresponds to which sample. I strongly feel that these data and complete metadata need to be deposited in a dedicated repository such as NCBI SRA archive.

Further, I have an issue with the use of controls. The authors do not explain how the controls were used in mosquito experiments. What is the background signal in qPCR assessment of viral infections? What is the likelihood that some of the virus-negative results for mosquito saliva samples were caused by the failure of saliva collection in the first place? Likewise, the authors mention negative controls in their microbiome study, but do not explain how they were used. Negative controls can be critically important for controlling the reagent and laboratory contamination, but the authors need to explain their use clearly, and ideally illustrate what they did.

Specific comments:

 Table 1 is not helpful if you do not provide information on how the samples were used. For example, 613 larvae collected in Albania in 2017 sound impressive enough — but were any of them used for any experiments/analyses at all?

 Please provide details of PCR reactions: reaction mix composition, cycling details

 Figure 2: I do not think that providing the full nucleotide sequence of mosquito COI gene is useful, and definitely not as the main-text figure. On a related note, despite this figure, I struggled to understand how specimens were selected for COI amplification, and which primers, and for which region, were ultimately used for the amplification. Please try to clarify these explanations.

 How was surface sterilization done?

 Figs 4-5: Two full-page figures showing multiple trees based on different-length regions of the COI gene are excessive - especially if similar results are also shown in a (more informative, in my view) network plot! Please decide on what way of showing these results would be the most useful for conveying your message. The current set of low-resolution trees, including many duplicates for site/sequence, and with multiple colored inserts, is not really accessible. 

 Table 3 and Figure 7: I have not found them informative or helpful. Move them to Supplement?

 Discussion of the microbiota: you focus on mammalian-pathogenic microbes, but please consider that many of those listed are known to live in a much broader spectrum of environments. For example, some strains of Bartonella are members of the healthy gut microbiota of honey bees, and about many other strains, we do not know!

Is the work clearly and accurately presented and does it cite the current literature?

Partly

If applicable, is the statistical analysis and its interpretation appropriate?

Yes

Are all the source data underlying the results available to ensure full reproducibility?

No

Is the study design appropriate and is the work technically sound?

Yes

Are the conclusions drawn adequately supported by the results?

Partly

Are sufficient details of methods and analysis provided to allow replication by others?

Partly

Reviewer Expertise:

Insect biodiversity and microbiome

We confirm that we have read this submission and believe that we have an appropriate level of expertise to state that we do not consider it to be of an acceptable scientific standard, for reasons outlined above.
